# Bacterial Profile among Patients with Suspected Bloodstream Infections in Ethiopia: A Systematic Review and Meta-Analysis

**DOI:** 10.1155/2020/8853053

**Published:** 2020-09-10

**Authors:** Birhan Alemnew, Habtamu Biazin, Asmamaw Demis, Melese Abate Reta

**Affiliations:** ^1^Department of Medical Laboratory Sciences, College of Health Sciences, Woldia University, Woldia, Ethiopia; ^2^Department of Microbiology, Immunology and Parasitology, School of Medicine, College of Health Sciences, Addis Ababa University, Addis Ababa, Ethiopia; ^3^Department of Nursing, College of Health Sciences, Woldia University, Woldia, Ethiopia; ^4^Department of Medical Microbiology, Faculty of Health Sciences, University of Pretoria, Pretoria, South Africa

## Abstract

**Introduction:**

The burden of bloodstream infections (BSIs) has been warranted in Ethiopia. Globally, the emergency and raised resistance rate of bacterial antimicrobial resistance is becoming a prominent problem, and it is difficult to treat patients having sepsis. In this review, we aimed to determine the pooled prevalence of bacterial isolates among presumptive patients with bloodstream infections in Ethiopia.

**Methods:**

A systematic search was performed from PubMed/MEDLINE, Scopus, HINARI, ScienceDirect, and Google Scholar electronic databases using PRISMA guidelines. The data analysis was carried out using STATA^TM^ version 14 after the records were cleaned and sorted out.

**Results:**

A total of 26 studies with 8,958 blood specimens and 2,382 culture-positive bacterial isolates were included for systematic review and meta-analysis. The meta-analysis derived a pooled culture-positive bacterial prevalence which was 25.78% (95% CI: 21.55–30.01%). The estimated pooled prevalence of Gram-positive and Gram-negative bacterial isolates was 15.50% (95% CI: 12.84–18.15%) and 10.48 % (95% CI: 8.32–12.63%), respectively. The two common Gram-positive bacteria isolated from patients suspected of BSIs were coagulase-negative *Staphylococcus* with a pooled prevalence of 5.75% (95% CI: 4.58–6.92%) and *S. aureus* 7.04 % (95% CI: 5.37–8.72%). Similarly, the common Gram-negative bacterial isolates and their estimated pooled prevalence were *E. coli* 1.69% (95% CI: 1.21–2.16%), *Klebsiella* species 7.04 % (95% CI: 5.37–8.72%), *Pseudomonas* species 0.39% (95% CI: 0.08–0.70%), *Salmonella* species 1.09% (95% CI: 0.79–1.38%), and *Streptococcus pyogenes* 0.88% (95% CI: 0.54–1.22%).

**Conclusion:**

The prevalence of bacterial isolates among presumptive patients suspected to BSIs in Ethiopia remains high. Furthermore, we found a remarkable variation in the pathogen distribution across the study setting.

## 1. Background

Bloodstream infections are the leading cause of morbidity and mortality throughout the world [1]. Globally, around 200,000 cases of BSIs with mortality of rates ranging from 20 to 50% were reported annually [2]. In low- and middle-income countries including Ethiopia, BSIs are a major public health concern and cause illnesses and deaths in all groups of population [3], especially in immunocompromised individuals such as patients in an intensive care unit (ICU), elders, children [4], cancer patients [5, 6], neonates [7], and patients living with human immunodeficiency virus (HIV) [8].

Bacterial bloodstream infections are defined as the presence of viable bacteria in the bloodstream that can elicit an immune response [9]. Bacteria may enter the bloodstream invasion, normally sterile parts of the body, in different ways. It is a serious, life-threatening infection that gets worse very quickly due to the spread of microorganisms and their toxins in blood [10]. Both Gram-negative and positive bacteria in a wide range of bacteria species cause BSIs [11]. As many previous studies highlighted, the common types of bacteria causing bloodstream infections are Gram-positive bacteria such as *Staphylococcus aureus*, coagulase-negative *Staphylococci* (CoNS), *Streptococcus pyogenes*, *Streptococcus pneumoniae*, *Streptococcus agalactiae*, and *Enterococcus faecium* and Gram-negative bacteria such as *Escherichia coli, Pseudomonas aeruginosa,* and *Klebsiella* species [4, 11, 12].

Blood is normally a sterile site, in which a blood culture has a high positive predictive value and is a key laboratory diagnostic tool/or specimen for an accurate bacteremia diagnosis [13]. Furthermore, blood culture is considered a highly sensitive test to identify such bacterial isolates that can cause bloodstream infection; also, it is easy to perform [14]. Thus, rapid detection and identification of these possible bacterial pathogens in blood culture are very essential, and the determination of their antimicrobial resistance profile has a key role to diminish the impact of bacteria associated with bloodstream infections [15].

Treatment of BSI is usually done by the timely administration of appropriate antimicrobial agents based on the sensitivity profile of the causative agents. However, due to the emergency and wide distribution of resistant bacteria to most of the currently prescribed antibiotics, it has become a serious health problem with many economic and social inferences all over the world [16].

Clinical disease caused by multidrug-resistant bacteria prolongs the duration of illness, hospital stays, and healthcare-associated costs and makes patients lose protection to invasive procedures; besides, it lowers productivity and harms the global economy [17].

On the contrary, bloodstream infections have an impact on dental prechemotherapy and transplant prescribed antimicrobial agents due to developing resistance; as a result, it is difficult to treat dental/oral health, transplantation, hematological diseases, and viral diseases such as coronavirus through reducing the immune systems and increasing the risk of morbidity and mortality due to underline BSIs [18–21].

Extensive use and misuse of antibiotics in clinical, environmental, and agricultural areas, empirical treatment, and taking the drug without prescription are the major contributors to the emergence of antimicrobial resistance (AMR) and the development of different antibiotic-resistant gene mutations [22–24]. Additionally, the global movement of people and the extensive use of antibiotics as the last sort of drug for extended-spectrum antibiotics give rise to carbapenem resistance [24, 25]. Furthermore, prolonged hospital stay, presence of underline medical conditions, and invasive procedures contribute to the rise of AMR [26].

The spread of drug resistance for many antibiotics makes the treatment options for bacterial bloodstream infections difficult. For this reason, in 2015, the World Health Organization (WHO) set strategies for detection, prevention, and control of AMR [27–29]. This strategy comprises improving awareness and understanding of AMR, performing surveillance and research to increase the knowledge and strengthen the evidence about AMR, improving the use of antimicrobial agents in human and animal health, and developing new drugs, vaccines, diagnostic tools, and other interventions in all countries [30, 31].

Despite many efforts that have been undergoing, many bacterial pathogens have become resistant to the most common antibiotics and become a serious public health concern with economic and social implications. Globally, antimicrobial resistance is a major challenge, particularly in resource-limited countries including Ethiopia. Even though timely and appropriate use of antibiotics is the only way to treat bacterial bloodstream infection, there are no strict antibiotic stewardship practices and information showing the countrywide pooled prevalence of bloodstream infection-causing bacterial isolates and their antimicrobial resistance patterns, which may halt the resistance pattern. Therefore, this systematic review and meta-analysis aimed to determine pooled estimates of the prevalence of bacterial isolates causing bloodstream infections among presumptive patients of bloodstream infections in Ethiopia. Besides, this will help as a benchmark for developing antimicrobial stewardship programs and generating evidence-based selection of antimicrobial agents for proper treatment.

## 2. Methods

### 2.1. Protocol and Registration

We strictly follow the Preferred Reporting Items for Systematic Review and Meta-Analyses (PRISMA) tool to report the findings of this systematic review and meta-analysis [32]. The completed PRISMA checklist is provided as a supplementary file (additional file: Table S1). The protocol for this study was submitted to the International Prospective Register of Systematic Reviews (PROSPERO) in February 2020 and was assigned the identification number (ID#149999).

### 2.2. Data Sources and Search Strategies

A systematic search was carried out using the following electronic databases: PubMed/Medline, HINARI, Scopus, ScienceDirect, and Google Scholar. Medical subject headings and related keywords were used extensively to search the appropriate articles from these databases using the following combinations of keywords: “bacterial isolates,” “bacterial pathogen,” “bacteria,” “bloodstream infection,” “bacteremia,” “sepsis,” “septicemia,” “antibiotic resistance pattern,” “antimicrobial resistance,” “antimicrobial susceptibility,” “antimicrobial susceptibility test,” and “Ethiopia.” These search words/phrases were further paired with each other or combined using “AND” and “OR” Boolean operators. Furthermore, reference lists of all included studies were screened to identify further potentially eligible studies and gray literature studies. Only those articles written in the English language and conducted in Ethiopia were considered.

### 2.3. Inclusion and Exclusion Criteria

Observational studies fulfilling the following criteria were included: (a) studies reporting bloodstream infections across all age groups and conducted in Ethiopia; (b) articles published in the English language and published from 2000 to 2020. Additionally, case reports, policy statements, reviews, and inaccessible full texts or those unable to receive from the corresponding author communicated through e-mail were excluded from the study.

### 2.4. Study Selection and Quality Assessment

All retrieved studies were exported into EndNote reference manager software version 8 (Thomson Reuters, London), and duplicated studies were removed. Four reviewers (BA, HB, AD, and MAR) independently screened the titles and abstracts, and full texts were reviewed to determine the eligibility of each study. Where there was disagreement, a decision was reached after discussion and consensus among all reviewers. On the contrary, the critical quality assessment checklist recommended by the “Joanna Briggs Institute (JBI)” was used to evaluate the quality of the included studies [33]. Two reviewers (BA and MAR) independently assessed the quality of the full-text articles. The discrepancy was resolved through discussion to reach on consensus and to include articles to the final analysis. The domain paper quality assessment criteria were clear inclusion criteria, details of study subjects, the study settings, reliable/valid measurements for exposure, outcome variables, and appropriate statistical analysis (additional file, Table S2). The cutoff point was declared after reviewing the relevant literature. Disagreements between the two authors were resolved by taking the mean score of the two authors' evaluations.

### 2.5. Data Extraction

All articles included in the final analysis were reviewed by two authors independently using standardized data extraction tools prepared in the Microsoft Excel sheet. The following data were extracted from each original article using the data abstraction form: author's name, year of publication, study region, study area, study design, study period, sample size, the prevalence of bacterial BSI, type of bacterial isolates, and prevalence of bacterial isolates.

### 2.6. Data Processing and Analysis

The extracted data using Microsoft Excel sheet 2016 were transferred into STATA version 14 software (Stata Corporation, College Station, Texas) for final analysis. Due to the presence of significant heterogeneity across studies, the random-effect model was applied to estimate the pooled effect size, odds ratios (ORs), and 95% confidence intervals (CIs) across studies. Subgroup analysis was conducted by sample size, year of publication, and study region. Heterogeneity of all included studies was assessed using the *I*^2^-statistical test. A *p* value of less than 0.05 was used to declare heterogeneity. Heterogeneity across the studies examined using the *I*^2^ statistics was categorized to 25%, 50%, and 75% which represent low, moderate, and high, respectively [34]. The source of heterogeneity was examined through sensitivity analysis and subgroup analysis. The presence of publication bias was evaluated using Egger's regression test with a *p* value of less than 0.05 as a cutoff point to declare the presence of publication bias [29].

## 3. Results

### 3.1. Search Results

**Figure 1:**
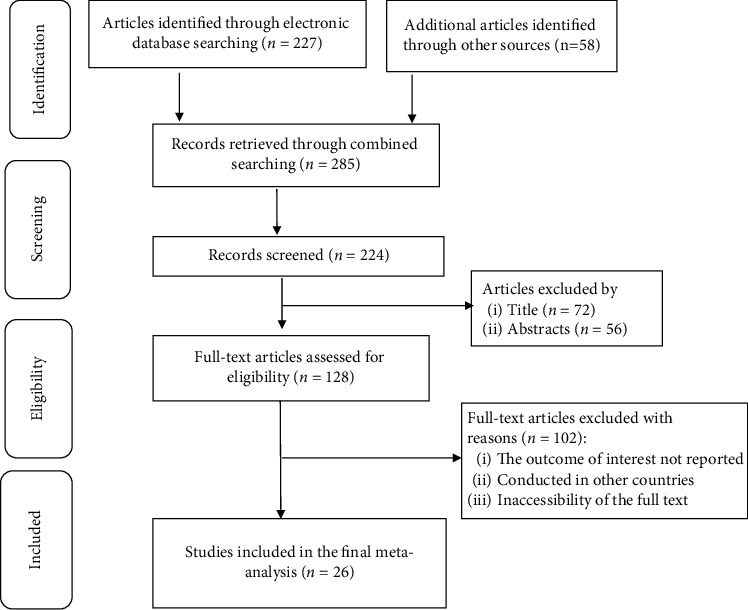
PRISMA-adapted flow diagram showing the results of the search and reasons for the exclusion of articles [29].

As illustrated in Figure 1, we identified a total of 285 potentially relevant studies from electronic databases, and 39 articles were excluded due to duplication. After reviewing the titles and abstracts, 128 articles were excluded because they did not meet the objectives and the inclusion criteria of the review. Accordingly, 128 full-text articles were reviewed in-depth based on the preset inclusion criteria, of which 102 articles were excluded with reasons. Finally, 26 studies were considered and used for the final quantitative analysis (meta-analysis).

### 3.2. Characteristics of Included Studies

As illustrated in Table 1, all the included studies in the final quantitative analysis were observational, 20 were cross-sectional [7, 14, 16, 35, 36, 38, 39, 42–47, 49–53, 55] and 6 were retrospective [11, 16, 40, 41, 48, 52] by study design. The included studies were conducted in four regions (Amhara, Tigray, Oromia, and Southern Nations, Nationalities, and Peoples' Region (SNNPR)) and from the two self-administrative cities (Addis Ababa and Dire Dawa). Of the 26 studies that fulfilled the review inclusion criteria [7, 11, 14, 16, 35, 36, 38–52, 55], eleven studies were conducted in Amhara region [7, 8, 11, 14, 36, 40, 44, 45, 52–54], nine in Addis Ababa [16, 41, 43, 46, 48–50, 55, 56], three in Oromia region [35, 38, 42], and a single study from Tigray [39], Dire Dawa [51], and SNNPR [47]. The sample size of individual studies ranged from 83 [53] to 856 [36].

### 3.3. Culture-Positive Bacterial Profile among Patients with Suspected Bloodstream Infections in Ethiopia

**Figure 2:**
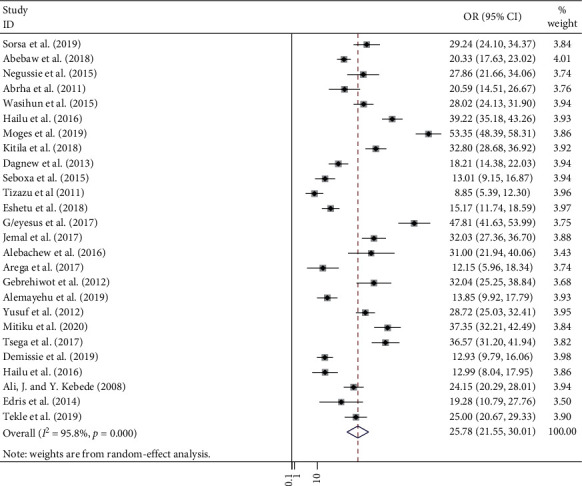
Forest plot showing the pooled prevalence of bacterial profile among patients with suspected bloodstream infections in Ethiopia.

**Figure 3:**
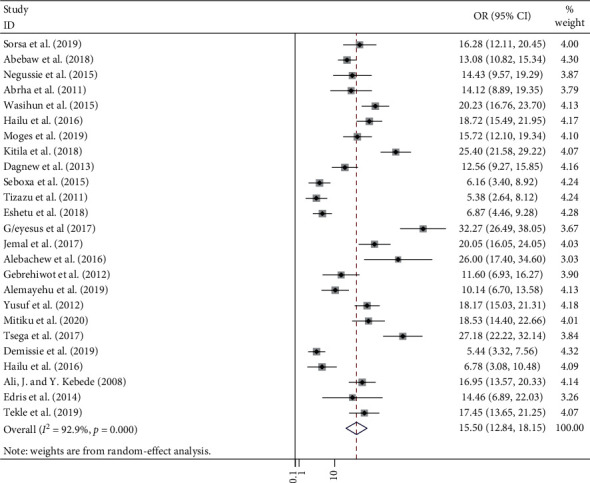
Forest plot showing the pooled prevalence of Gram-positive bacterial isolates among patients with suspected bloodstream infections in Ethiopia.

**Figure 4:**
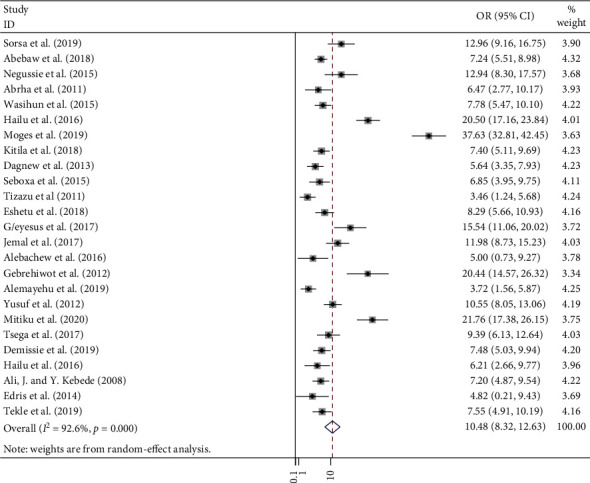
Forest plot showing the pooled prevalence of Gram-negative bacterial isolates among patients with suspected bloodstream infections in Ethiopia.

**Figure 5:**
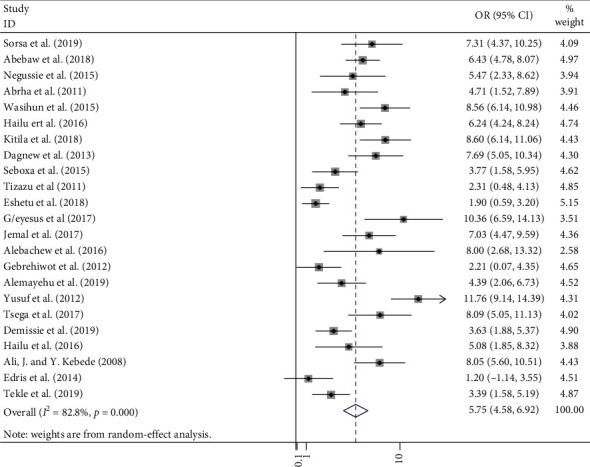
Forest plot showing the pooled prevalence CoNS bacterial isolates among patients with suspected bloodstream infections in Ethiopia.

**Figure 6:**
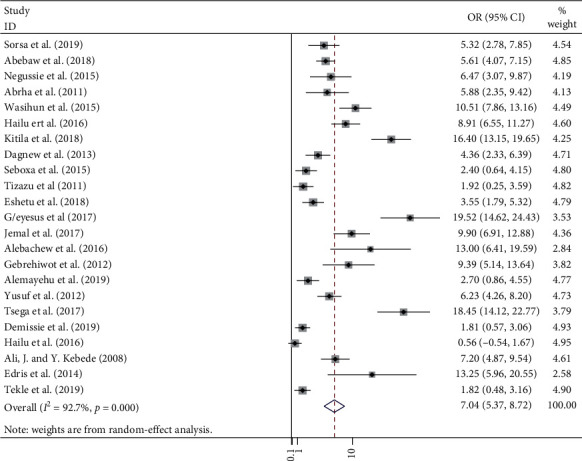
Forest plot showing the pooled prevalence of *S. aureus* isolated among patients with suspected bloodstream infections in Ethiopia.

In this meta-analysis, a total of 2,382 positive bacteria cultures obtained from 8,958 blood samples were included. The meta-analysis-derived pooled culture-positive bacterial prevalence from all blood samples was 25.78 % (95% CI: 21.55–30.01%) (Figure 2). Pooled prevalence of Gram-positive and Gram-negative bacterial isolates was 15.50 % (95% CI: 12.84–18.15%) (Figure 3) and 10.48 % (95% CI: 8.32–12.63%) (Figure 4), respectively. The two common Gram-positive bacteria among patients suspected for bloodstream infections were recovered. The pooled prevalence of CoNS was 5.75 % (95% CI: 4.58–6.92%) (Figure 5). Similarly, the pooled estimated prevalence of *S. aureus* from these groups was 7.04 % (95% CI: 5.37–8.72%) (Figure 6). Likewise, we found five Gram-negative bacterial isolates among patients suspected for bloodstream infection in Ethiopia. The pooled estimates of *Klebsiella* species isolates were found to be 7.04 % (95% CI: 5.37–8.72%) (additional file: Figure S1 and Table 2) followed by *E. coli 1*.69% (95% CI: 1.21–2.16%) (additional file: Figure S2 and Table 2), *Salmonella* species 1.09% (95% CI: 0.79–1.38%) (additional file: Figure S3 and Table 2), *S. pyogenes* 0.88% (95% CI:0.54–1.22%) (additional file: Figure S4 and Table 2), and *Pseudomonas* species 0.39% (95% CI: 0.08–0.70%) (additional file: Figure S5 and Table 2).

### 3.4. Heterogeneity and Publication Bias

The existence of heterogeneity and publication bias was assessed within the included studies. Consequently, there was considerable heterogeneity across twenty six included studies (*I*^2^ = 95.8%). Egger's regression test for publication bias revealed marginally significant (*p*=0.044), which indicated the presence of publication bias (additional file: Figure S6). Moreover, heterogeneity across studies considered for Gram-positive isolates was *I*^2^ = 92.9% and Gram-negative isolates was *I*^2^ = 92.6%. The publication bias of Gram-positive and Gram-negative bacterial isolates was found to be *p*=0.001 and *p*=0.001, respectively, using Egger's regression test.

### 3.5. Subgroup Analysis

Due to the presence of high heterogeneity across or within the included studies, we conducted subgroup analysis based on the study area (region), sample size, and year of publication to sort out the possible source of heterogeneity across the studies. However, the subgroup analysis result revealed that the source of heterogeneity was not due to the study region, sample size, and year of publication disparities (Table 3 and Supplementary Figures S7–S9).

### 3.6. Sensitivity Analysis

The sensitivity analysis showed that the effect of individual studies on the pooled estimate was insignificant, suggesting the robustness of the aggregated estimate. Therefore, the pooled prevalence of bacterial isolates was steady and reliable when examined by neglecting one study at a time (additional file: Figure S10).

## 4. Discussion

To our knowledge, the result of this review documented that the pooled prevalence of bacterial isolates causing bloodstream infections in Ethiopia remains high. The overall pooled prevalence of BSIs by bacterial isolates from blood cultures was 27.78%. The finding was relatively lower as compared to findings from meta-analysis done in West Africa in which the pooled prevalence of BSIs was 31.70% [57]. On the contrary, our finding was higher than a study done in low- and middle-income countries that the prevalence of bacterial isolates from community-acquired pediatric bloodstream infection was 19.1% [58]. Moreover, previous studies reported that bacterial positive blood culture ranged from 7 to 13.9% [59, 60]. Similarly, a systematic review done in Africa reported that the pooled prevalence of bacterial isolates from blood specimens among bloodstream infections was 17.4% [61]. Moreover, significant bloodstream infections and antibiotic resistance in the ICU were observed in North India, in which the blood culture positivity was estimated at 12% [62]. Bacterial isolates in a blood sample with a pooled prevalence of 7.4% were reported in Harare, Zimbabwe [63]. Furthermore, a previous systematic review and meta-analysis study done in Africa and Asia region indicated that the median prevalence of BSIs was 12.50% [64]. The possible explanation for the discrepancies might be due to the drug stewardship program, geographical location, epidemiological difference of the etiological agents, and nature of the patients.

The current review revealed that the pooled prevalence of Gram-positive bacterial isolates was 15.50 %. A study conducted in resource-limited countries revealed that the prevalence of Gram-positive bacterial isolates in the blood culture was relatively lower, 6.2% [59].

Similarly, in this meta-analysis, the pooled prevalence of Gram-negative bacterial isolates was 10.48 %. This finding was relatively similar to the previously conducted systematic review report which revealed that the pooled prevalence of Gram-negative bacterial isolates in blood specimens from children was 7.7% [59].

Furthermore, the rapid rise of bacterial bloodstream infections and evidence of resistance to commonly prescribed antimicrobial agents have been a warranted public health problem in prevention and treatment of oral health/dental care transplantation process, cancer chemotherapy, hematological diseases, and others due to presensitized and existence of the resistance gene and suppressed host immune systems as a result of bacterial BSIs [18–21].

### 4.1. Limitations of the Study

Our review had many limitations. First, there was no documented history of antimicrobial therapy and history of antibiotic intake. Second, in this study, only the English language articles were considered for the analysis. Third, due to the lack of documented antibiogram data, we were unable to review and evaluate BSI causing bacteria multidrug resistance profile. Last, antimicrobial susceptibility standards and interpretive criteria change over time, which may have variations in interpretations and findings.

## 5. Conclusions

Generally, in this meta-analysis, we found a wide variety of bacterial isolates with the high pooled prevalence of both Gram-negative and Gram-positive bacteria, in particular *S. aureus,* CoNS*, Klebsiella* species*, E. coli, S. pyogenes, Salmonella* species, and *Pseudomonas* species. Therefore, strengthening of the tool to diagnose BSIs should be a routine practice to detect pathogenic bacteria in blood to select the appropriate/better antibiotics to treat the bacteria causing BSIs. Besides, an effort should be given to the resistance pattern, molecular genetics to detect specific resistance gene mutations associated with different antibiotics, and characterizations of its phylogenetic parameters for those commonly identified bacteria l isolates causing bloodstream infections.

## Figures and Tables

**Table 1:** Characteristics of included studies in this meta-analysis.

Authors	Year	Region	Study area	Hospital unit	Specimen	Study design	Study period	Sample size	Number of isolates (*n*)	Gram-positive bacterial isolates, *n* (%)	Gram-negative bacterial isolates, *n* (%)
Sorsa et al. [35]	2019	Oromia	ASRH	Neonatal sepsis	Blood	Cross-sectional	April 2016–May 2017	301	88 (29.24%)	49 (16.28%)	39 (12.96%)
Abebaw Shiferaw et al. [36]	2018	Amhara	UoGTH	Bacteremia-suspected patients	Blood	Retrospective	September 2003–February 2013	856	174 (20.33%)	112 (13.08%)	62 (7.24%)
Negussie et.al. [37]	2015	Addis Ababa	TASH and YH	Septicemia-suspected children	Blood	Cross-sectional	October 2011–February 2012	201	56 (27.86%)	29 (14.43%)	26 (12.94%)
Abrha et al. [38]	2011	Oromia	JURH	Malnourished children admitted	Blood	Cross-sectional	October 2009–May 2010	170	35 (20.59%)	24 (14.12%)	11 (6.47%)
Wasihun et al. [39]	2015	Tigray	MK	Outpatients	Blood	Cross-sectional	March–October 2014	514	144 (28.02%)	104 (20.23%)	40 (7.78%)
Hailu et al. [14]	2016	Amhara	BRHLC	Outpatients	Blood	Cross-sectional	March 2013–January 2015	561	220 (39.22%)	105 (18.72%)	115 (20.50%)
Moges et al. [40]	2019	Amhara	FCSH	Bacteremia-suspected patients	Blood	Cross-sectional	December 2017–April 2018	388	207 (53.35%)	61 (15.72%)	146 (37.63%)
Terfa Kitila et al. [41]	2018	Addis Ababa	AARL	Bacteremia-suspected patients	Blood	Retrospective	January 2015–December 2016	500	164 (32.8%)	127 (25.4%)	37 (7.40%)
Dagnew et al. [11]	2013	Amhara	UoGTH	Septicemia-suspected patients	Blood	Retrospective	September 2006–January 2012	390	71 (18.21%)	49 (12.56%)	22 (5.64%)
Seboxa et al. [16]	2015	Addis Ababa	TASH	Septicemia-suspected patients	Blood	Retrospective	August 2012–October 2013	292	38 (13.04%)	18 (6.16%)	20 (6.85%)
Tizazu et al. [42]	2011	Oromia	JUSH	Septicemia-suspected patients	Blood	Cross-sectional	October 2009–March 2010	260	23 (8.85%)	14 (5.39%)	9 (3.46%)
Eshetu et al. [43]	2018	Addis Ababa	TASH	Septicemia-suspected patients	Blood	Cross-sectional	September 2016–October 2017	422	64 (15.17%)	29 (6.88%)	35 (8.29%)
Geyesus et al. [44]	2017	Amhara	UoGTH	UoGTH Suspected of neonatal sepsis	Blood	Cross-sectional	September 2015–May 2016	251	120 (47.81%)	81 (32.27%)	39 (15.54%)
Jemal [8]	2017	Amhara	FURH	BSI in HIV-infected patients	Blood	Cross-sectional	December 2016	384	123 (32.03%)	77 (20.05%)	46 (12%)
Alebachew et al. [45]	2016	Amhara	UoGTH	Patients infected with HIV and suspected of having sepsis	Blood	Cross-sectional	March–May 2013	100	31 (31%)	26 (26%)	5 (5%)
Arega et al. [46]	2017	Addis Ababa	TASH	Adult febrile cancer patients	Blood	Cross-sectional	December 2011–June 2012	107	13 (12.15%)	-	-
Gebrehiwot et al. [7]	2012	Amhara	UoGTH	Clinically suspected neonatal sepsis	Blood	Cross-sectional	July 2011–June 2012	181	58 (32.04%)	21 (11.60%)	37 (20.44%)
Alemayehu et al. [47]	2019	SNNP	HUCSH	Pediatric patients	Blood	Cross-sectional	March–August 2016	296	41 (13.85%)	30 (10.14%)	11 (3.72%)
Adib and Worku [48]	2012	Addis Ababa	TASH	Suspected of neonatal sepsis	Blood	Retrospective	September 1, 2007–August 31, 2008	578	166 (28.72%)	105 (18.17%)	61 (10.55%)
Mitiku et al. [49]	2019	Addis Ababa	TASH	Febrile pediatric patients	Blood	Cross-sectional	September 2017–June 2018	340	127 (37.35%)	63 (18.53%)	74 (21.76%)
Tsega et al. [50]	2017	Addis Ababa	ZMH	Septicemia-suspected pediatric patients	Blood	Cross-sectional	June 2016–March 2017	309	113 (36.57%)	84 (27.18%)	29 (9.39%)
Demissie et al. [51]	2019	Dire Dawa	DCRH	Sepsis-suspected women attending the delivery	Blood	Cross-sectional	May–July 30, 2019	441	57 (12.9%)	24 (5.44%)	33 (7.48%)
Hailu et al. [14]	2016	Addis Ababa	TASH	Septicemia-suspected patients	Blood	Cross-sectional	January–March 2014	177	23 (12.99%)	12 (6.78%)	11 (6.22%)
Ali and Kebede [52]	2008	Amhara	UoGTH	Febrile patients	Blood	Retrospective	March 2001–April 2005	472	114 (24.15%)	80 (16.95%)	34 (7.20%)
Endris et al. [53]	2014	Amhara	UoGTH	Confirmed VL patients suspected of sepsis	Blood	Cross-sectional	February 2012–May 2012	83	16 (19.28%)	12 (14.46%)	4 (4.82%)
Gebre-Egziabher et al. [54]	2019	Amhara	UoGTH	ICU patients suspected of septicemia	Blood	Cross-sectional	February–May 2018	384	96 (25%)	67 (17.45%)	29 (7.55%)

ATRH: Asella Teaching and Referral Hospital, UoGTH: University of Gondar Teaching Hospital, TASH: Tikur Anbessa Specialized Hospital, YH: Yekatit 12 Hospital, TUSH: Jimma University Specialized Hospital, MK: Mekelle Hospital, BRHRLC: Bahir Dar Regional Health Research Laboratory Center, FHCSH: Felege Hiwot Comprehensive Specialized Hospital, AARL: Addis Ababa Regional Laboratory, GUH: Gondar University Hospital, HUCSH: Hawassa University Comprehensive Specialized Hospital, ZMH: Zewuditu Memorial Hospital, and DCRH: Dil Chora Referral Hospital.

**Table 2:** Results of each bacterial type isolated from bloodstream-infected patients.

Type of bacterial isolates	No. of studies	Total no. of culture-positive/from blood total specimens	Pooled prevalence rate (95% CI)	*I* ^2^ (*p* values)
*S.pyrogenes* [36, 38, 39, 41, 42, 44, 45, 47]	8	31/2,947	0.88 (0.54, 1.22)	0.0% (<0.638)

*Pseudomonas* species [7, 11, 14, 36, 39–41, 43, 45, 48, 51]	8	59/5,108	0.39 (0.08, 0.70)	0.0% (<0.885)

*Salmonella* species [7, 11, 36–38, 40, 42, 48, 51, 52, 56]	11	7/4,628	1.09 (0.79, 1.38)	51.1% (<0.021)

*E*. *coli* [7, 11, 14, 35, 36, 38–45, 47, 48, 50–54, 56]	21	156/7,634	1.69 (1.21, 2.16)	65.9% (<0.001)

*Klebsiella* species [7, 8, 11, 14, 35–45, 47, 48, 50–54, 56]	23	362/8,219	4.30 (2.45, 6.16)	91.0% (<0.001)

**Table 3:** Subgroup pooled prevalence analysis of bacterial isolates among patients suspected of bloodstream infection in Ethiopia, 2020.

Subgroup by	Characteristics	No. of studies	Total sample size	Pooled prevalence rate (95% CI)	Heterogeneity*I*^2^ (*p* values)
Study area	Oromia [35, 38, 42]	3	731	19.45 (6.41, 32.48)	95.5% (<0.001)
	Amhara [7, 8, 11, 14, 36, 40, 44, 45, 52–54]	11	4,050	31.11 (24.28, 37.95)	95.8% (<0.001)
	Addis Ababa [16, 41, 43, 46, 48–50, 55, 56]	9	2,926	24.05 (17.27, 30.83)	95.0% (<0.001)
	Others^*∗*^ [39, 47, 51]	3	1,251	18.23 (8.88, 27.58)	99.0% (<0.001)

Sample size	>300 [8, 11, 35, 36, 39–41, 43, 48–52, 54]	14	6,840	26.76 (23.62, 33.90)	96.0% (<0.001)
	≤300 [7, 14, 16, 37, 38, 42, 44–47, 53, 56]	12	2,118	21.53 (14.92, 28.14)	94.1% (<0.001)

Publication year	2000–2014 [7, 11, 38, 42, 48, 52, 53]	7	2,134	21.59 (15.27, 27.90)	82.9% (<0.001)
	2015–2020 [8, 14, 16, 35–41, 43–47, 49–51, 54]	19	6,824	27.31 (22.02, 32.60)	96.4% (<0.001)

Overall		26	8,958	25.78 (21.55, 30.01)	95.8% (<0.001)

^*∗*^Others: Tigray, Southern Nations, Nationalities, and Peoples' Region, and Dire Dawa.

## Data Availability

All generated data and research materials used during this systematic review and meta-analysis are available from the corresponding author upon reasonable request.

## Abbreviations

BSIs: Bloodstream infections
CI: Confidence interval
HIV: Human immunodeficiency virus
MeSH: Medical subject headings
MOOSE: Meta-analysis of observational studies in epidemiology
AMR: Antimicrobial resistance
NOS: Newcastle-Ottawa scale
OR: Odds ratio
PRISMA: Preferred Reporting Items for Systematic Reviews and Meta-Analyses
PROSPERO: International Prospective Register of Systematic Reviews
WHO: World Health Organization.

## Supplementary Materials

Supplementary Materials The data (PRISMA check list and additional findings) are available in the supplementary file.
