# Supplementary material for: Bacterial Profile among Patients with Suspected Bloodstream Infections in Ethiopia: A Systematic Review and Meta-Analysis
Source: Int J Microbiol. 2020 Sep 10;2020:8853053. doi: 10.1155/2020/8853053 (PMC7501548; doi:10.1155/2020/8853053)
Supplement: Supplementary Materials — The data (PRISMA check list and additional findings) are available in the supplementary file. [file 8853053.f1.zip › 8853053.f1/additonal file 1 docx.docx]

# Figures

NOTE: Weights are from random effects analysis

Overall (I-squared = 91.0%, p = 0.000)

Abebaw et al. (2018)

Yusuf et al. (2012)

Alemayehu et al. (2019)

Abrha et al. (2011)

Kitila et al. (2018)

Eshetu et al. (2018)

Alebachew et al. (2016)

Tekle et al. (2019)

ID

Hailu ert al. (2016)

Edris et al. (2014)

Jemal et al. (2017)

Hailu et al. (2016)

G/eyesus et al (2017)

Sorsa et al. (2019)

Study

Mahdi, J. and Y. Kebede (2008)

Moges et al. (2019)

Tizazu et al (2011)

Wasihun et al. (2015)

Dagnew et al. (2013)

Gebrehiwot et al. (2012)

Demissie et al. (2019)

Tsega et al. (2017)

Negussie et.al (2015)

4.30 (2.45, 6.16)

1.29 (-0.81, 3.39)

2.77 (-0.22, 5.76)

1.69 (-0.79, 4.17)

3.53 (-0.04, 7.10)

4.60 (0.91, 8.29)

2.61 (-0.38, 5.60)

1.00 (-0.95, 2.95)

9.38 (4.58, 14.18)

OR (95% CI)

6.24 (2.29, 10.19)

2.41 (-0.60, 5.42)

5.73 (1.59, 9.87)

2.82 (-0.39, 6.03)

7.57 (2.72, 12.42)

3.65 (0.12, 7.18)

2.12 (-0.59, 4.83)

23.20 (20.21, 26.19)

1.15 (-0.92, 3.22)

0.19 (-0.67, 1.05)

2.31 (-0.53, 5.15)

5.52 (1.15, 9.89)

1.36 (-0.86, 3.58)

6.80 (2.26, 11.34)

4.48 (0.52, 8.44)

100.00

4.73

4.45

4.62

4.24

4.20

4.45

4.77

3.76

Weight

4.10

4.45

4.02

4.38

3.74

4.26

%

4.55

4.45

4.74

4.98

4.50

3.93

4.70

3.87

4.10

4.30 (2.45, 6.16)

1.29 (-0.81, 3.39)

2.77 (-0.22, 5.76)

1.69 (-0.79, 4.17)

3.53 (-0.04, 7.10)

4.60 (0.91, 8.29)

2.61 (-0.38, 5.60)

1.00 (-0.95, 2.95)

9.38 (4.58, 14.18)

6.24 (2.29, 10.19)

2.41 (-0.60, 5.42)

5.73 (1.59, 9.87)

2.82 (-0.39, 6.03)

7.57 (2.72, 12.42)

3.65 (0.12, 7.18)

2.12 (-0.59, 4.83)

23.20 (20.21, 26.19)

1.15 (-0.92, 3.22)

0.19 (-0.67, 1.05)

2.31 (-0.53, 5.15)

5.52 (1.15, 9.89)

1.36 (-0.86, 3.58)

6.80 (2.26, 11.34)

4.48 (0.52, 8.44)

100.00

4.73

4.45

4.62

4.24

4.20

4.45

4.77

3.76

Weight

4.10

4.45

4.02

4.38

3.74

4.26

%

4.55

4.45

4.74

4.98

4.50

3.93

4.70

3.87

4.10

0

.1

1

10

Fig.S1: Forest plot showed the pooled prevalence of Klebsiella species isolated among patients with suspected bloodstream infections in Ethiopia

NOTE: Weights are from random effects analysis

Overall (I-squared = 65.9%, p = 0.000)

Edris et al. (2014)

Gebrehiwot et al. (2012)

Alemayehu et al. (2019)

Dagnew et al. (2013)

Study

Abrha et al. (2011)

Tsega et al. (2017)

G/eyesus et al (2017)

Eshetu et al. (2018)

Hailu et al. (2016)

Tekle et al. (2019)

Moges et al. (2019)

Yusuf et al. (2012)

Hailu ert al. (2016)

Demissie et al. (2019)

Tizazu et al (2011)

Kitila et al. (2018)

ID

Wasihun et al. (2015)

Alebachew et al. (2016)

Abebaw et al. (2018)

Sorsa et al. (2019)

Mahdi, J. and Y. Kebede (2008)

1.69 (1.21, 2.16)

1.20 (-1.14, 3.55)

3.31 (0.71, 5.92)

0.68 (-0.26, 1.61)

1.28 (0.17, 2.40)

0.59 (-0.56, 1.74)

1.62 (0.21, 3.02)

4.78 (2.14, 7.42)

0.95 (0.02, 1.87)

0.56 (-0.54, 1.67)

1.82 (0.48, 3.16)

3.09 (1.37, 4.82)

1.04 (0.21, 1.86)

3.39 (1.89, 4.88)

2.95 (1.37, 4.53)

1.54 (0.04, 3.03)

1.20 (0.25, 2.15)

OR (95% CI)

3.11 (1.61, 4.61)

1.00 (-0.95, 2.95)

1.75 (0.87, 2.63)

5.98 (3.30, 8.66)

0.42 (-0.16, 1.01)

100.00

2.71

2.34

6.21

5.62

%

5.52

4.75

2.30

6.24

5.66

4.94

3.92

6.56

4.49

4.28

4.50

6.14

Weight

4.48

3.42

6.39

2.25

7.28

1.69 (1.21, 2.16)

1.20 (-1.14, 3.55)

3.31 (0.71, 5.92)

0.68 (-0.26, 1.61)

1.28 (0.17, 2.40)

0.59 (-0.56, 1.74)

1.62 (0.21, 3.02)

4.78 (2.14, 7.42)

0.95 (0.02, 1.87)

0.56 (-0.54, 1.67)

1.82 (0.48, 3.16)

3.09 (1.37, 4.82)

1.04 (0.21, 1.86)

3.39 (1.89, 4.88)

2.95 (1.37, 4.53)

1.54 (0.04, 3.03)

1.20 (0.25, 2.15)

3.11 (1.61, 4.61)

1.00 (-0.95, 2.95)

1.75 (0.87, 2.63)

5.98 (3.30, 8.66)

0.42 (-0.16, 1.01)

100.00

2.71

2.34

6.21

5.62

%

5.52

4.75

2.30

6.24

5.66

4.94

3.92

6.56

4.49

4.28

4.50

6.14

Weight

4.48

3.42

6.39

2.25

7.28

0

.1

1

10

Fig.S2: Forest plot showed the pooled prevalence of *E.Coli* isolated among patients with suspected bloodstream infections in Ethiopia

Overall (I-squared = 51.1%, p = 0.021)

Moges et al. (2019)

Gebrehiwot et al. (2012)

Wasihun et al. (2015)

ID

Demissie et al. (2019)

Hailu et al. (2016)

Dagnew et al. (2013)

Mahdi, J. and Y. Kebede (2008)

Abrha et al. (2011)

Study

Tizazu et al (2011)

Yusuf et al. (2012)

Abebaw et al. (2018)

Negussie et.al (2015)

1.09 (0.79, 1.38)

0.52 (-0.20, 1.23)

3.31 (0.71, 5.92)

1.95 (0.75, 3.14)

OR (95% CI)

1.81 (0.57, 3.06)

0.56 (-0.54, 1.67)

0.77 (-0.10, 1.64)

3.39 (1.76, 5.02)

1.76 (-0.21, 3.74)

0.38 (-0.37, 1.14)

1.56 (0.55, 2.57)

1.17 (0.45, 1.89)

1.49 (-0.18, 3.17)

100.00

17.47

1.30

6.22

Weight

5.72

7.27

11.79

3.33

2.26

%

15.66

8.70

17.11

3.16

1.09 (0.79, 1.38)

0.52 (-0.20, 1.23)

3.31 (0.71, 5.92)

1.95 (0.75, 3.14)

1.81 (0.57, 3.06)

0.56 (-0.54, 1.67)

0.77 (-0.10, 1.64)

3.39 (1.76, 5.02)

1.76 (-0.21, 3.74)

0.38 (-0.37, 1.14)

1.56 (0.55, 2.57)

1.17 (0.45, 1.89)

1.49 (-0.18, 3.17)

100.00

17.47

1.30

6.22

Weight

5.72

7.27

11.79

3.33

2.26

%

15.66

8.70

17.11

3.16

0

.1

1

10

Fig.S3: Forest plot showed the pooled prevalence of *Salmonella Species* isolated among patients with suspected bloodstream infections in Ethiopia

Overall (I-squared = 0.0%, p = 0.638)

G/eyesus et al (2017)

Kitila et al. (2018)

ID

Alebachew et al. (2016)

Alemayehu et al. (2019)

Tizazu et al (2011)

Wasihun et al. (2015)

Abrha et al. (2011)

Abebaw et al. (2018)

Study

0.88 (0.54, 1.22)

2.39 (0.50, 4.28)

0.60 (-0.08, 1.28)

OR (95% CI)

2.00 (-0.74, 4.74)

1.01 (-0.13, 2.15)

1.15 (-0.14, 2.45)

0.97 (0.12, 1.82)

1.76 (-0.21, 3.74)

0.70 (0.14, 1.26)

100.00

3.18

24.79

Weight

1.51

8.73

6.74

15.78

2.90

36.37

%

0.88 (0.54, 1.22)

2.39 (0.50, 4.28)

0.60 (-0.08, 1.28)

2.00 (-0.74, 4.74)

1.01 (-0.13, 2.15)

1.15 (-0.14, 2.45)

0.97 (0.12, 1.82)

1.76 (-0.21, 3.74)

0.70 (0.14, 1.26)

100.00

3.18

24.79

Weight

1.51

8.73

6.74

15.78

2.90

36.37

%

0

.1

1

10

Fig. S4: Forest plot showed the pooled prevalence of *S.pyogen*es isolated among patients with suspected bloodstream infections in Ethiopia

Overall (I-squared = 0.0%, p = 0.885)

Hailu ert al. (2016)

Moges et al. (2019)

Abebaw et al. (2018)

Eshetu et al. (2018)

Gebrehiwot et al. (2012)

Hailu et al. (2016)

ID

Dagnew et al. (2013)

Demissie et al. (2019)

Kitila et al. (2018)

Alebachew et al. (2016)

Yusuf et al. (2012)

Wasihun et al. (2015)

Study

0.39 (0.08, 0.70)

2.67 (-2.57, 7.91)

1.29 (-1.24, 3.81)

0.70 (-0.67, 2.07)

0.95 (-0.91, 2.81)

2.76 (-2.65, 8.18)

1.69 (-1.63, 5.02)

OR (95% CI)

1.03 (-0.98, 3.04)

1.36 (-1.31, 4.03)

0.40 (-0.38, 1.18)

1.00 (-0.96, 2.96)

1.21 (-1.16, 3.58)

0.19 (-0.19, 0.58)

100.00

0.35

1.50

5.06

2.77

0.33

0.87

Weight

2.36

1.34

15.54

2.49

1.70

65.70

%

0.39 (0.08, 0.70)

2.67 (-2.57, 7.91)

1.29 (-1.24, 3.81)

0.70 (-0.67, 2.07)

0.95 (-0.91, 2.81)

2.76 (-2.65, 8.18)

1.69 (-1.63, 5.02)

1.03 (-0.98, 3.04)

1.36 (-1.31, 4.03)

0.40 (-0.38, 1.18)

1.00 (-0.96, 2.96)

1.21 (-1.16, 3.58)

0.19 (-0.19, 0.58)

100.00

0.35

1.50

5.06

2.77

0.33

0.87

Weight

2.36

1.34

15.54

2.49

1.70

65.70

%

0

.1

1

10

Fig. S5: Forest plot showed the pooled prevalence of Pseudomonas Species isolated among patients with suspected bloodstream infections in Ethiopia

Fig. S6: funnel plots showing publication bias of included studies, p (prevalence) of bacterial isolates represented in the x-axis and SE (standard error) in the y-axis


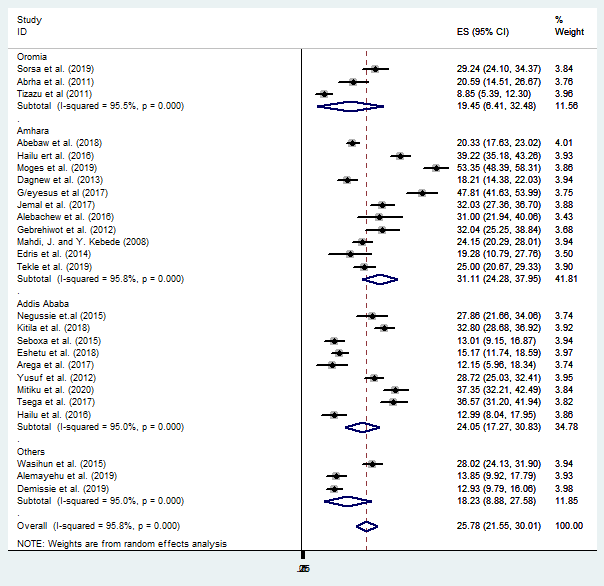
NB: Others: South nation nationality people, Tigray, Dire Dawa

Fig. S7. Subgroup analysis based on study area/region for the pooled prevalence of bacterial isolates causing blood stream infection in Ethiopia, 2020


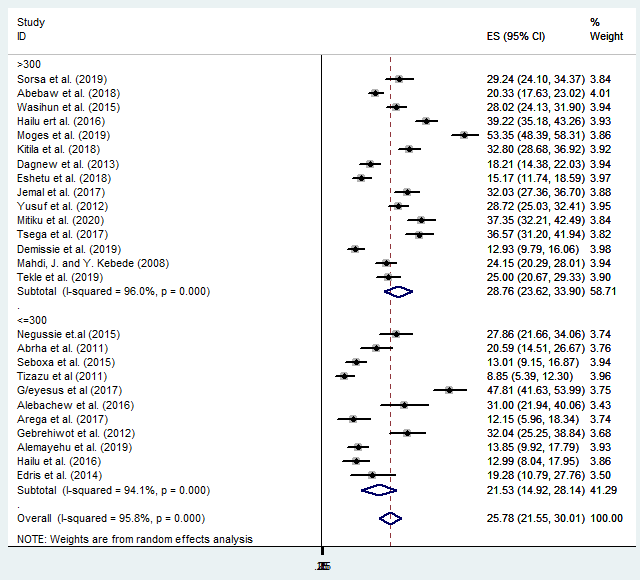


Fig S8. Subgroup analysis based on sample size for the pooled prevalence of bacterial isolates causing blood stream infection in Ethiopia, 2020.


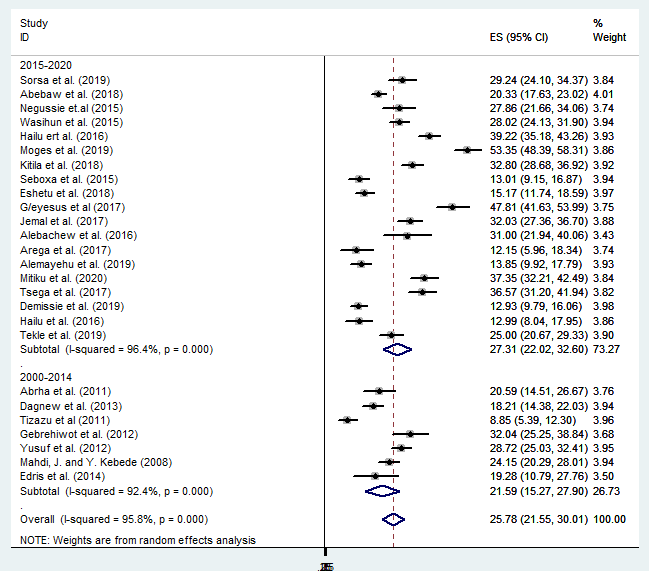


Fig. S9. Subgroup analysis based on year publication for the pooled prevalence of bacterial isolates causing blood stream infection in Ethiopia, 2020


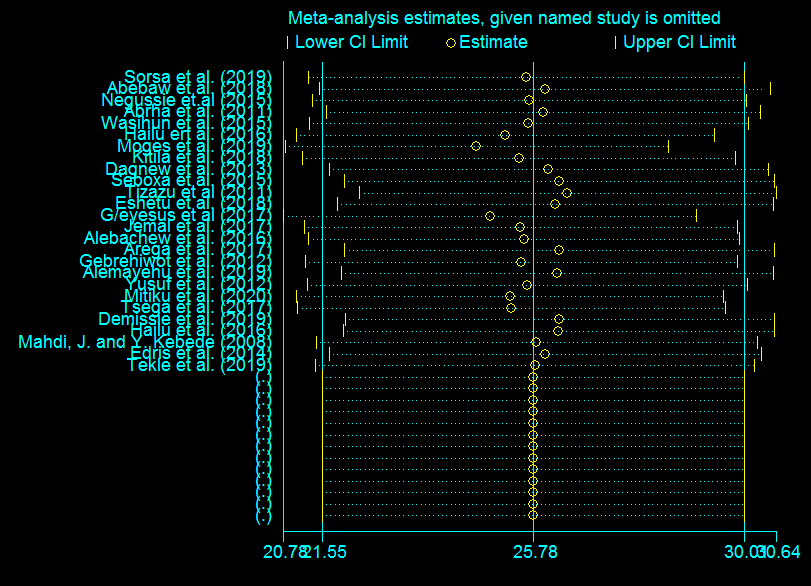


Fig. S10: Sensitivity analysis pooled prevalence pooled prevalence of bacterial isolates causing blood stream infection in Ethiopia
