# Supplementary material for: Bacterial Profile among Patients with Suspected Bloodstream Infections in Ethiopia: A Systematic Review and Meta-Analysis
Source: Int J Microbiol. 2020 Sep 10;2020:8853053. doi: 10.1155/2020/8853053 (PMC7501548; doi:10.1155/2020/8853053)
Supplement: Supplementary Materials — The data (PRISMA check list and additional findings) are available in the supplementary file. [file 8853053.f1.zip › 8853053.f1/Table S2_Quality assessment of included studies.docx]

**Table S2:** Quality appraisal result of included studies; Using Joanna Briggs Institute (JBI) quality appraisal checklist for cross-sectional studies (1).

| Author/s and year | **1) Were Inclusion Criteria Clearly Defined?** | **2) Details of study subjects and the setting/s?** | **3) Reliable/valid measurement for exposure** | **4) Standard measurement for the condition** | **5) Were confounding factors identified?** | **6) Were strategies to deal cofounding factors stated?** | **7) Reliable/valid measurement for outcome** | **8) Appropriate Statistical analysis** | **Quality items met (n/8)** |
| --- | --- | --- | --- | --- | --- | --- | --- | --- | --- |
| Sorsa et al., 2019 (2) | YES | YES | YES | YES | UN | NA | YES | YES | 6/8 |
| Abebaw et al., 2018 (3) | YES | YES | YES | YES | UN | NA | YES | YES | 6/8 |
| Negussie et.al., 2015(4) | YES | YES | YES | YES | UN | NA | YES | YES | 6/8 |
| Abrha et al., 2011 (5) | YES | YES | YES | YES | UN | NA | YES | YES | 6/8 |
| Wasihun et al., 2015 (6) | YES | YES | YES | YES | UN | NA | YES | YES | 6/8 |
| Hailu ert al., 2016(7) | YES | YES | YES | YES | UN | NA | YES | YES | 6/8 |
| Moges et al.2019(8) | YES | YES | YES | YES | UN | NA | YES | YES | 6/8 |
| Kitila et al., 2018(9) | YES | YES | YES | YES | UN | YES | YES | YES | 7/8 |
| Dagnew et al., 2013(10) | YES | YES | YES | YES | UN | NA | YES | YES | 6/8 |
| Seboxa et al., 2015(11) | YES | YES | YES | YES | UN | NA | YES | YES | 6/8 |
| Tizazu et al., 2011(12) | YES | YES | YES | YES | YES | NA | YES | YES | 7/8 |
| Eshetu et al., 2018(13) | YES | YES | YES | YES | UN | NA | YES | YES | 6/8 |
| G/eyesus et al., 2017(14) | YES | YES | YES | YES | YES | NA | YES | YES | 7/8 |
| Jemal et al., 2017(15) | YES | YES | YES | YES | UN | NA | YES | YES | 6/8 |
| Alebachew et al., 2016(16) | YES | YES | YES | YES | UN | NA | YES | YES | 6/8 |
| Arega et al., 2017(17) | YES | YES | YES | YES | UN | NA | YES | YES | 6/8 |
| Gebrehiwot et al., 2012(18) | YES | YES | YES | YES | UN | NA | YES | YES | 6/8 |
| Alemayehu et al., 2019(19) | YES | YES | YES | YES | UN | NA | YES | YES | 6/8 |
| Yusuf et al., 2012(20) | YES | YES | YES | YES | UN | NA | YES | YES | 6/8 |
| Mitiku et al., 2019(21) | YES | YES | YES | YES | UN | NA | YES | YES | 6/8 |
| Tsega et al., 2017(22) | YES | YES | YES | YES | UN | NA | YES | YES | 6/8 |
| Demissie et al., 2019(23) | YES | YES | YES | YES | UN | NA | YES | YES | 6/8 |
| Hailu et al., 2016(7) | YES | YES | YES | YES | UN | NA | YES | YES | 6/8 |
| Mahdi, J. and Y. Kebede, 2008(24) | YES | YES | YES | YES | UN | NA | YES | YES | 6/8 |
| Edris et al., 2014(25) | YES | YES | YES | YES | UN | NA | YES | YES | 6/8 |
| Tekle et al., 2019(26) | YES | YES | YES | YES | UN | NA | YES | YES | 6/8 |

NA = not applicable, UN=Unclear.

**References**

1. Joanna Briggs Institute. The Joanna Briggs Institute Critical Appraisal tools for use in JBI Systematic Reviews: Checklist for Prevalence Studies. Retreived November. 2017;15:2018.

2. Sorsa A, Fruh J, Stotter L, Abdissa S. Blood culture result profile and antimicrobial resistance pattern: a report from neonatal intensive care unit (NICU), Asella teaching and referral hospital, Asella, south East Ethiopia. Antimicrob Resist Infect Control. 2019;8:42.

3. Abebaw Shiferaw AA, Tesera H, Belachew T, Mihiretie GD. The bacterial profile and antibiotic susceptibility pattern among patients with suspected bloodstream infections, Gondar, north-west Ethiopia. Pathology and Laboratory Medicine International. 2018;Volume 10:1-7.

4. Negussie A, Mulugeta G, Bedru A, Ali I, Shimeles D, Lema T, et al. Bacteriological Profile and Antimicrobial Susceptibility Pattern of Blood Culture Isolates among Septicemia Suspected Children in Selected Hospitals Addis Ababa, Ethiopia. International journal of biological and medical research. 2015;6(1):4709-17.

5. Abrha A, Abdissa A, Beyene G, Getahun G, Girma T. Bacteraemia among severely malnourished children in jimma university hospital, ethiopia. Ethiop J Health Sci. 2011;21(3):175-82.

6. Wasihun AG, Wlekidan LN, Gebremariam SA, Dejene TA, Welderufael AL, Haile TD, et al. Bacteriological profile and antimicrobial susceptibility patterns of blood culture isolates among febrile patients in Mekelle Hospital, Northern Ethiopia. SpringerPlus. 2015;4:314.

7. Hailu D, Abera B, Yitayew G, Mekonnen D, Derbie A. Bacterial blood stream infections and antibiogram among febrile patients at Bahir Dar Regional Health Research Laboratory Center, Ethiopia. Ethiopian Journal of Science and Technology. 2016;9(2):103.

8. Moges F, Eshetie S, Abebe W, Mekonnen F, Dagnew M, Endale A, et al. High prevalence of extended-spectrum beta-lactamase-producing Gram-negative pathogens from patients attending Felege Hiwot Comprehensive Specialized Hospital, Bahir Dar, Amhara region. PloS one. 2019;14(4):e0215177.

9. Terfa Kitila K, Taddese BD, Hailu TKm, Sori LM, mariam K, Geleto SE, et al. Assessment of Bacterial Profile and Antimicrobial Resistance Pattern of Bacterial Isolates from Blood Culture in Addis Ababa Regional Laboratory, Addis Ababa, Ethiopia. Clinical Microbiology: Open Access. 2018;07(02).

10. Dagnew M, Yismaw G, Gizachew M, Gadisa A, Abebe T, Tadesse T, et al. Bacterial profile and antimicrobial susceptibility pattern in septicemia suspected patients attending Gondar University Hospital, Northwest Ethiopia. BMC Res Notes. 2013;6(1):283.

11. Seboxa T, Amogne W, Abebe W, Tsegaye T, Azazh A, Hailu W, et al. High Mortality from Blood Stream Infection in Addis Ababa, Ethiopia, Is Due to Antimicrobial Resistance. PloS one. 2015;10(12):e0144944.

12. Zenebe T, Kannan S, Yilma D, Beyene G. Invasive Bacterial Pathogens and their Antibiotic Susceptibility Patterns in Jimma University Specialized Hospital, Jimma, Southwest Ethiopia. Ethiop J Health Sci. 2011;21(1):1-8.

13. Seneshat Eshetu, Adane Bitew, Tigist Getachew, Abera D, Gizaw S. Multi-Drug Resistance Profile of Bacteria Isolated from Blood Stream Infection at Tikur Anbessa Specialized Hospital, Addis Ababa, Ethiopi. 2018.

14. G/Eyesus, Moges F, Eshetie S, Yeshitela B, Abate E. Bacterial etiologic agents causing neonatal sepsis and associated risk factors in Gondar, Northwest Ethiopia. BMC Pediatr. 2017;17(1):137.

15. Jemal M. Bacterial Bloodstream Infections and Their Antimicrobial Susceptiblity Pattern Among Hiv/Aids Patients At Felege Hiwot Referral Hospital, Bahir Dar, Amahara Regional State, North West Ethiopia: Addis Ababa University; 2017.

16. Alebachew G, Teka B, Endris M, Shiferaw Y, Tessema B. Etiologic Agents of Bacterial Sepsis and Their Antibiotic Susceptibility Patterns among Patients Living with Human Immunodeficiency Virus at Gondar University Teaching Hospital, Northwest Ethiopia. Biomed Res Int. 2016;2016:5371875.

17. Arega B, Wolde-Amanuel Y, Adane K, Belay E, Abubeker A, Asrat D. Rare bacterial isolates causing bloodstream infections in Ethiopian patients with cancer. Infect Agent Cancer. 2017;12:40.

18. Amare Gebrehiwot, Wubishet Lakew, Feleke Moges, Beyene Moges, Belay Anagaw, Gizachew Yismaw, et al. Bacterial profile and drug susceptibility pattern of neonatal sepsis in Gondar University Hospital, Gondar northwest Ethiopia. 2018.

19. Alemayehu T, Tadesse E, Ayalew S, Nigusse B, Yeshitila B, Amsalu A, et al. HIGH BURDEN OF NOSOCOMIAL INFECTIONS CAUSED BY MULTI-DRUG RE-SISTANT PATHOGENS IN PEDIATRIC PATIENTS AT HAWASSA UNIVERSITY COMPREHENSIVE SPECIALIZED HOSPITAL. 2019.

20. Adib Yusuf, Worku B. Descriptive cross –sectional study on neonatal sepsis in the Neonatal intensive care unit of Tekur Anbessa Hospital, Addis Ababa , Ethiopia. 2012.

21. Mequanint Mitiku, Zeleke Ayenew, Desta K. Multi-drug resistant, extended spectrum beta-lactamase and carbapenemase producing bacterial isolates among children under five years old with suspected bloodstream infection in a specialized hospital in Ethiopia: Cross-sectional study. 2019.

22. Tsega D. Bacterial profile, antimicrobial susceptibility pattern and associated risk Factors among septicemia suspected pediatrics patients at Zewuditu Memorial Hospital, Addis Ababa, Ethiopia: Addis Ababa University; 2017.

23. Daniel Demissie, Berhanu Seyoum, Melake Demena, Biruk Yeshitila. EPTICEMIA, BACTERIAL ISOLATES AND DRUG SUSCEPTIBILITY AMONG WOMEN ATTENDING DELIVERY AT DILCHORA HOSPITAL, DIRE DAWA, EASTERN ETHIOPIA. 2019.

24. Mahdi J, Kebede Y. Frequency of isolation and antimicrobial susceptibility pattern of bacterial isolates from blood culture, Gondar University teaching hospital, Northwest Ethiopia. Ethiopian medical journal. 2008;46:155-61.

25. Endris M, Takele Y, Woldeyohannes D, Tiruneh M, Mohammed R, Moges F, et al. Bacterial sepsis in patients with visceral leishmaniasis in Northwest Ethiopia. Biomed Res Int. 2014;2014:361058.

26. Tekle Gebre-egziabher FMY, Zemene Tigabu Kebede, Alem Getaneh Mehari Bacterial profile and their antibiotic resistance patterns from blood culture in Intensive Care Unit patients at the University Of Gondar Comprehensive Specialized Hospital, Northwest Ethiopia. 2019;PREPRINT (Version 1) available at Research Square [+<https://doi.org/10.21203/rs.2.9515/v1>+.
