# Supplementary material for: Bacterial Profile among Patients with Suspected Bloodstream Infections in Ethiopia: A Systematic Review and Meta-Analysis
Source: Int J Microbiol. 2020 Sep 10;2020:8853053. doi: 10.1155/2020/8853053 (PMC7501548; doi:10.1155/2020/8853053)
Supplement: Supplementary Materials — The data (PRISMA check list and additional findings) are available in the supplementary file. [file 8853053.f1.zip › 8853053.f1/Table S1. PRISMA 2009 checklist.doc]

| **Section/topic** | **#** | **Checklist item** | **Reported on page #** |
| --- | --- | --- | --- |
| **TITLE** | | |  |
| Title | 1 | Bacterial profile among patients with suspected bloodstream infections in Ethiopia: A systematic review and Meta-analysis | 01 |
| **ABSTRACT** | | |  |
| Structured summary | 2 | **Background**: The burden of blood stream infections(BSIs) has been warranted in Ethiopia. Globally, the emergency and raised resistance rate of bacterial antimicrobial resistance is becoming a prominent problem and it is difficult to treat patients having sepsis. In this review, we aimed to determine the pooled prevalence of bacterial isolates among presumptive patients with bloodstream infections in Ethiopia. **Methods:** A systematic search was performed from PubMed/MEDLINE, SCOPUS, HINARI, Science Direct, and Google Scholar electronic databases. The data analysis was carried out using STATA^TM^ after the records were cleaned and sorted out. The random-effects model was applied to estimate the pooled effect size, odds ratios (ORs), and 95% confidence intervals (CIs) across studies. Subgroup analysis was conducted by sample size, year of publication, and study region. The I^2^ statistical test was used to check heterogeneity between the studies, and the publication bias was evaluated using the funnel plot and the egger’s regression test.  **Results:** A total of 26 studies with 8,958 blood specimens, and 2,382 culture-positive bacterial isolates were included for systematic review and meta-analysis. The meta-analysis derived a pooled culture-positive bacterial prevalence was 25.78 % (95%Cl: 21.55- 30.01%). The estimated pooled prevalence of gram-positive and gram-negative bacterial isolates was 15.50% (95%Cl: 12.84,-18.15%), and 10.48 % (95%Cl: 8.32-12.63%), respectively. The two common gram-positive bacteria isolated from patients suspected of BSIs were coagulase-negative staphylococcus with a pooled prevalence of 5.75% (95%Cl: 4.58-6.92%), and *S. aureus* 7.04 % (95%Cl: 5.37-8.72%). Similarly, the common gram-negative bacterial isolates and their estimated pooled prevalence were *E. Coli1.*69% (95%Cl: 1.21-2.16%), Klebsiella Species 7.04 % (95%Cl: 5.37-8.72%), Pseudomonas Species 0.39% (95%Cl: 0.08- 0.70%), Salmonella Species 1.09% (95%Cl: 0.79-1.38%), and *Streptococcus pyogenes* 0.88% (95%Cl:0.54-1.22%).  **Conclusion**: The prevalence of bacterial isolates among presumptive patients suspected to BSIs in Ethiopia remains high. Also, we found a remarkable variation in pathogen distribution and their resistance profile across the study setting. A strengthened antimicrobial resistance profile surveillance and adherence to a strict antimicrobial stewardship program are warranted to reduce the burden of the disease. | 02 |
| **INTRODUCTION** | | |  |
| Rationale | 3 | Bloodstream infections are the leading cause of morbidity and mortality throughout the world (1). Globally, around 200, 000 cases of BSI with mortality of rates ranging from 20-50% were reported annually (2). In developing nation including Ethiopia BSI is a major public health concern and cause illnesses and death in all people of ages (3), especially in immunocompromised individuals such as patients in an intensive care unit (ICU), elders, and children(4), cancer patients (5, 6), neonates (7), and patients living with Human Immunodeficiency Virus (HIV) (8)  Bacterial bloodstream infections are defined as the presence of viable bacteria in the bloodstream which can elicit an immune response (9). Bacteria may enter the bloodstream, invasion normally sterile parts of the body in different ways. It is a serious, life-threatening infection that gets worse very quickly due to the spread of microorganisms and their toxins in the blood (10). Both gram-negative and positive bacteria in a wide range of bacteria species cause BSI (11). As many previous studies highlighted, the common type bacteria causing bloodstream infections are gram-positive such as *Staphylococcus aureus*, *coagulase-negative staphylococci* (CoNS), *Streptococcus pyogenes*, *Streptococcus pneumoniae*, *Streptococcus agalactiae*, and *Enterococcus faecium* and gram-negative bacteria such as *Escherichia coli, Pseudomonas aeruginosa,* Klebsiella species (4, 11, 12). | 04 |
| Objectives | 4 | This systematic review and meta-analysis aimed to determine pooled estimates of the prevalence of bacterial isolates causing bloodstream infection among presumptive patients of bloodstream infections in Ethiopia. | 04 |
| **METHODS** | | |  |
| Protocol and registration | 5 | The protocol for this study was submitted to the International Prospective Register of Systematic Reviews (PROSPERO) on February 2020, and has been assigned the identification number (ID#149999). | 05 |
| Eligibility criteria | 6 | Observational studies fulfilling the following criteria were included. (a) Studies reporting the bloodstream infection and/or antimicrobial resistance pattern and/or its associated factors across all age groups conducted in Ethiopia; (b) articles published in the English language, and published from 2000 to 2020. Additionally, Case reports, policy statements, reviews, and inaccessible full texts or unable to receive from the corresponding author communicated through email were excluded from the study. | 05 |
| Information sources | 7 | A systematic search was carried out using the following electronic databases: PubMed/Medline, HINARI, Scopus, Sciences Direct, and Google Scholar electronic databases. | 05 |
| Search | 8 | Medical subject heading and related keywords were used extensively to search the appropriate articles from these databases using the following combinations of keywords: “bacterial isolates”, “bacterial pathogen”, "bacteria", “bloodstream infection”, "bacteremia", "sepsis", "septicemia", “antibiotic resistance pattern”, “antimicrobial resistance”, “antimicrobial susceptibility”, "antimicrobial susceptibility test”, and "Ethiopia". These search words/phrases were further paired with each other or combined using “AND” and “OR” Boolean operators. Also, reference lists of all included studies were screened to identify further potentially eligible studies and gray literature. Only those articles written in the English language and conducted in Ethiopia were considered. | 05 |
| Study selection | 9 | All retrieved studies were exported into EndNote reference manager software version 8 (Thomson Reuters, London), and duplicated studies were removed. Three reviewers (BA, HB, and AD) independently screened the titles and abstracts, and full texts were reviewed to determine the eligibility of each study. Where there was disagreement, a decision was reached after discussion and consensus among all reviewers. on the other hand, the critical quality assessment checklist recommended by the “Joanna Briggs Institute (JBI) was used to evaluate the quality of included studies (30). Two reviewers (BA and MA) independently assessed the quality of the full-text articles. The discrepancy was resolved through discussion to reach on consciences and to include articles to final analysis. The domain paper quality assessment criteria were; clear inclusion criteria, details of study subjects, and the study settings, reliable/valid measurements for exposure, outcome variables, and appropriate statistical analysis (Table S1). This cut-off point was declared after reviewing the relevant literature. Disagreements between the two authors were resolved by taking the mean score of the two author's evaluations. | 05 |
| Data collection process | 10 | All articles included in the final analysis were reviewed by two authors independently using standardized data extraction tools prepared in Microsoft excel sheet. The following data were extracted from each original article using this data abstraction form: author’s name, year of publication, study region, study area, study design, study period, sample size, the prevalence of bacterial BSI, type of bacterial isolates, and prevalence of bacterial isolates. | 06 |
| Data items | 11 | the prevalence of bacterial BSI, type of bacterial isolates and prevalence of bacterial isolates | 06 |
| Risk of bias in individual studies | 12 | Heterogeneity across the studies examined using the I^2^ statistics was categorized to 25%, 50%, and 75% which representing low, moderate, and high, respectively (31). The source of heterogeneity was examined through sensitivity analysis and subgroup analyses. The presence of publication bias was checked using the Egger's regression test with a p-value of less than 0.05 as a cut of point to declare the presence of publication bias (29). | 06 |
| Summary measures | 13 | The prevalence of bacterial BSI, type of bacterial isolates and prevalence of bacterial isolates | 06 |
| Synthesis of results | 14 | D The extracted data using Microsoft Excel sheet 2016 was transferred into STATA version 14 (Stata Corporation, College Station, Texas) software for final analysis. Due to the presence of significant heterogeneity across studies, the random-effects model was applied to estimate the pooled effect size, odds ratios (ORs), and 95% confidence intervals (CIs) across studies. Subgroup analysis was conducted by sample size, year of publication, and study region. Heterogeneity of all included studies was assessed using the I^2^ -statistical test. A p-value of less than 0.05 was used to declare heterogeneity. | 06 |

Page 1 of 2

| **Section/topic** | **#** | **Checklist item** | **Reported on page #** |
| --- | --- | --- | --- |
| Risk of bias across studies | 15 | The presence of publication bias was checked using the Egger's regression test with a p-value of less than 0.05 as a cut of point to declare the presence of publication bias (29). | 6 |
| Additional analyses | 16 | Subgroup analysis was conducted by sample size, year of publication, and study region. Heterogeneity of all included studies was assessed using the I^2^ -statistical test. | 06 |
| **RESULTS** | | |  |
| Study selection | 17 | As demonstrated in figure 1, we identified a total of 285 potentially relevant studies from electronic databases, 39 articles were excluded due to duplication. After reviewing the titles and abstracts, 128 articles were excluded because they did not meet the objectives and the inclusion criteria of this review. Accordingly, 128 full-text articles were reviewed in-depth based on the pre-set inclusion criteria of which, 102 articles were excluded with reasons. Finally, 26 studies were considered and used for final quantitative analysis (meta-analysis). | 07 |
| Study characteristics | 18 | As illustrated in Table 1, all the included studies in the final quantitative analysis were observational, 20 were cross-sectional (7, 14, 16, 33-49), and 6 were retrospective (11, 16, 50-53) by study design. The included studies were conducted in four regions (Amhara, Tigray, Oromia, and South Nation Nationality of People (SNNP)), and from the two self-administrative cities (Addis Ababa and Dire Dawa). Of the 26 studies met the review inclusion criteria (7, 11, 14, 16, 33-48, 50-53), eleven studies were conducted in Amhara region (7, 8, 11, 14, 34-36, 49-51, 54), nine in Addis Ababa (14, 16, 38-42, 52, 53), three in Oromia region (43-45), a single study from Tigray (46), Dire Dawa (47), and SNNP (48). The sample size of individual studies ranged from 83 (49) to 856 (34). | 08 |
| Risk of bias within studies | 19 | The existence of heterogeneity and publication bias was determined within the included studies. Consequently, there was considerable heterogeneity across thirty-seven included studies (I^2^ = 95.8%). | 14 |
| Results of individual studies | 20 | Similarly, the pooled estimated prevalence of *S. aureus* from these groups was 7.04 % (95%Cl: 5.37-8.72%) (Fig.6). Likewise, we found around five gram-negative bacterial isolates among patients suspected of bloodstream infection in Ethiopia. The pooled estimates of Klebsiella species isolates were found to be 7.04 % (95%Cl: 5.37- 8.72%) (Fig.S1. &Table 2), followed by *E. Coli 1*.69% (95%Cl: 1.21-2.16%) (Fig.S2. &Table 2), Salmonella species 1.09% (95%Cl: 0.79-1.38%) (Fig.S3& Table 2), *S.pyogenes* 0.88% (95%Cl:0.54-1.22%)(Fig.S4.& Table 2), and Pseudomonas species 0.39% (95%Cl: 0.08-0.70%)(Fig. S5& Table 2.). | 14-15 |
| Synthesis of results | 21 | In this meta-analysis, a total of 2,382 positive bacteria cultures obtained from 8,958 blood samples were included. The meta-analysis derived a pooled culture-positive bacterial prevalence from all blood samples was 25.78 % (95%Cl: 21.55-30.01%) (Fig. 2). Pooled prevalence of gram-positive and gram-negative bacterial isolates was 15.50 % (95%Cl: 12.84-18.15% (Fig.3), and 10.48 % (95%Cl: 8.32-12.63%) (Fig.4), respectively. The two common gram-positive bacteria among patients suspected bloodstream infections were recovered. The pooled prevalence of CoNS was 5.75 % (95%Cl: 4.58-6.92%) (Fig.5). | 07-14 |
| Risk of bias across studies | 22 | Egger’s regression test for publication bias revealed marginally significant (p=0.044), which indicated the presence of publication bias (Fig. S6). Moreover, heterogeneity across studies considered for gram-positive isolates was (I^2^ =92.9%, and gram-negative isolates were (I^2^ 92.6%). publication bias of gram-positive and gram-negative bacterial isolates was found to be p=0.001 and p=0.001, respectively using Egger’s regression test. | 13 |
| Additional analysis | 23 | Due to the presence of high heterogeneity across or within included studies, we conducted subgroup analysis based on study area (region), sample size, and year of publication to sort out the possible source of heterogeneity across the studies. But the subgroup analysis result revealed that the source of heterogeneity was not due to the study region, sample size, and year publication disparities (Table 3 and Fig 7-9.). | 14 |
| **DISCUSSION** | | |  |
| Summary of evidence | 24 | To our knowledge, the result of this review demonstrated that the pooled prevalence of bacterial isolates causing bloodstream infections in Ethiopia. The overall pooled prevalence of BSIs bacterial isolates from blood cultures was 27.78%. The finding was relatively lower as compared to findings from meta-analysis done in West Africa, in which the pooled prevalence of BSI was 31.70% (55). On the other hand, our finding was higher than a study done in low- and middle-income countries, the prevalence of bacterial isolates were 19.1% in community-acquired pediatric bloodstream infection (56). Also, previous studies reported that bacterial positive blood culture was ranged from 7 to 13.9% (57, 58). similarly, a systematic review done in Africa reported that the pooled prevalence of bacterial isolates from blood specimen among bloodstream infection was 17.4% (59). Moreover, significant bloodstream infections and antibiotic resistance in ICU in North India was observed, that the blood culture positivity was estimated at 12% (60). Bacterial isolates in a blood sample with a pooled prevalence of 7.4% were reported in Harare, Zimbabwe, 2012-2017 (61). Furthermore, a previous systematic review and meta-analysis study done in Africa and Asia region indicated that the median prevalence of BSIs was 12.50% (62). The possible explanation for the discrepancies might be due to the drug stewardship program, geographical location, epidemiological difference of the etiological agents, and nature of the patients.  The current review revealed that the pooled prevalence of gram-positive bacterial isolates was 15.50 %. A study conducted in resource-limited countries showed a relatively lower prevalence of gram-positive bacterial isolates in blood culture was 6.2% (57).  Similarly, in this meta-analysis, the pooled prevalence of gram-negative bacterial isolates was 10.48 %. The finding was relatively similar to previously reported data, a systematic review of antibiotics resistance among gram-negative bacteria in children with sepsis in resource-limited countries was reported as 7.7% (57). | 15-16 |
| Limitations | 25 | our review had many limitations. First, there was no documented history of antimicrobial therapy and previous history of antibiotics intake. Second, in this study, only English articles were considered for the analysis. Third, due to the lack of documented antibiogram data, we are unable to review and evaluate BSI causing bacteria's multidrug resistance profile. Last, antimicrobial susceptibility standards and interpretive criteria change over time, which may have variations in interpretations and findings. | 15 |
| Conclusions | 26 | Generally, in this meta-analysis, we found a wide variety of bacterial isolates with high pooled prevalence both gram-negative and gram-positive, in particular *S.aureus, CONs,* Klebesiella species*, Ecoli, S.pyogens,* Salmonella species, *and* Pseudomonas Species*.* Therefore, a strengthen the tool to diagnosis should be a routine practice to detect pathogenic bacteria in blood, to select the appropriate/better antibiotics to treat the bacteria cause BSIs. Furthermore, research priority should be given on-resistance pattern, molecular genetics to detect the specific resistance gene mutations associated with different antibiotics, characterizations of its phylogenetic parameter are warranted for these commonly identified bacteria which causing bloodstream infection. | 16 |
| **FUNDING** | | |  |
| Funding | 27 | NA | None |

*From:* Moher D, Liberati A, Tetzlaff J, Altman DG, The PRISMA Group (2009). Preferred Reporting Items for Systematic Reviews and Meta-Analyses: The PRISMA Statement. PLoS Med 6(7): e1000097. doi:10.1371/journal.pmed1000097

For more information, visit: **www.prisma-statement.org**.

Page 2 of 2
